# IADL Limitations Linked to Mortality and Cognitive Impairment in Older Puerto Rican Adults

**DOI:** 10.1093/geroni/igaf122.2150

**Published:** 2025-12-31

**Authors:** Caitlin Northcutt, Tyler Bell, Sadaf Milani, Michael Crowe

**Affiliations:** University of Kentucky, Lexington, Kentucky, United States; University of California San Diego, San Diego, California, United States; University of Texas Medical Branch, Galveston, Texas, United States; University of Alabama at Birmingham, Birmingham, Alabama, United States

## Abstract

Limitations in instrumental activities of daily living (IADL) are an important clinical indicator of dementia severity and risk factors for mortality. Research suggests that IADL limitations are a predictor of future cognitive decline. A greater understanding of this longitudinal association is needed, especially in underrepresented samples with a high risk of Alzheimer’s disease, such as older adults in Puerto Rico. This study examined these associations using data from Waves 1 (2002/2003), 2 (2006/2007), 3 (2020/2021), and 4 (2022/2024) of the Puerto Rican Elder: Health Conditions Study (PREHCO), a longitudinal, population-based study of Puerto Rican adults aged 60 and older. The sample included adults who were cognitively unimpaired at baseline (Mini Mental Cabán score of 11 or higher) with at least one wave of follow-up data (n = 2,630, mean age=71.33, SD = 8.25; 59.5% female). Participants self-reported limitations on seven IADLs and were screened for cognitive impairment at each wave. National Death Index data was integrated. Overall, increased baseline IADL limitations were associated with an increased risk of death [hazards ratio (HR)=1.05, p<.001], as were increases in IADL limitations over time (HR = 1.04, p<.001). When accounting for this mortality risk using inverse-probability weighting, increases in IADL limitations remained significantly related to a heightened risk of cognitive impairment (HR = 1.08, p<.001). In contrast, baseline IADL limitations did not (p=.102). Exploratory analyses revealed that these associations were moderated by sex and education. Findings highlight the need for early screening and interventions tailored to older Puerto Rican adults’ specific needs and lived experiences to sustain functional independence in mid-life.

